# Experimental Model Exposed to Bisphenol and Submitted to a High-Fat Diet and Related Morphologic Testicular Parameters Alterations Analysis

**DOI:** 10.1590/S1677-5538.IBJU.2024.9911

**Published:** 2024-05-20

**Authors:** Jorge Luiz Alves Pereira, Luiz Carlos Schrotke Pires, Eliete Dalla Corte Frantz, Dangelo Carlo Magliano, Luciano Alves Favorito

**Affiliations:** 1 Instituto de Educação Médica Rio de Janeiro RJ Brasil Instituto de Educação Médica - IDOMED - Campus Città América, Rio de Janeiro, RJ, Brasil;; 2 Departamento de Morfologia Centro de Pesquisa em Morfologia e Metabolismo Universidade Federal Fluminense Niterói RJ Brasil Departamento de Morfologia, Centro de Pesquisa em Morfologia e Metabolismo, Universidade Federal Fluminense – UFF, Niterói, RJ, Brasil;; 3 Unidade de Pesquisa Urogenital Universidade do Estado do Rio de Janeiro Rio de Janeiro RJ Brasil Unidade de Pesquisa Urogenital - Universidade do Estado do Rio de Janeiro - UERJ, Rio de Janeiro, RJ, Brasil

**Keywords:** Obesity, Testis, bisphenol S [Supplementary Concept]

## Abstract

**Purpose:**

To evaluate the morphological and stereological parameters of the testicles in mice exposed to bisphenol S and/or high-fat diet-induced obesity.

**Material and Methods:**

Forty adult male C57BL/6 mice were fed a standard diet (SC) or high-fat diet (HF) for a total of 12 weeks. The sample was randomly divided into 4 experimental groups with 10 mices as follows: a) SC - animals fed a standard diet; b) SC-B - animals fed a standard diet and administration of BPS (25 μg/kg of body mass/day) in drinking water; c) HF: animals fed a high-fat diet; d) HF-B - animals fed a high-fat diet and administration of BPS (25 μg/Kg of body mass/day) in drinking water. BPS administration lasted 12 weeks, following exposure to the SC and HF diets. BPS was diluted in absolute ethanol (0.1%) and added to drinking water (concentration of 25 μg/kg body weight/day). The animals were euthanized, and the testes were processed and stained with hematoxylin and eosin (H&E) for morphometric and stereological parameters, including density of seminiferous tubules per area, length density and total length of seminiferous tubules, height of the tunica albuginea and the diameter of the seminiferous tubules. The images were captured with an Olympus BX51 microscope and Olympus DP70 camera. The stereological analysis was done with the Image Pro and Image J programs. Means were statistically compared using ANOVA and the Holm-Sidak post-test (p<0.05).

**Results:**

The seminiferous tubule density per area reduced in all groups when compared with SC samples (p<0.001): HF (40%), SC-B 3(2%), and HF-B (36%). Length density was reduced significantly (p<0.001) in all groups when compared with SC group: HF (40%), SC-B (32%), and HF-B (36%). The seminiferous tubule total length was reduced (p<0.001) when compared to f HF (28%) and SC-B (26%) groups. The tubule diameter increased significantly (p<0.001) only when we compared the SC group with SC (54%) an SC-B (25%) groups and the tunica thickness increased significantly only in HF group (117%) when compared with SC-B (20%) and HF-B 31%.

**Conclusion:**

Animals exposed to bisphenol S and/or high-fat diet-induced obesity presented important structural alterations in testicular morphology.

## INTRODUCTION

Currently, obesity is described as a global health problem and characterized by the excessive accumulation of body fat (1). Its prevalence has almost tripled since 1975, with around 13% of adults obese, and will continue to increase rapidly in the coming years, it is estimated that 1 in 5 adults will be obese by the year 2025, with that number increasing to more than 40% of the global population by 2030 and of which more than 200 million are men (1-3). Obesity has been linked to a variety of medical conditions, including hormonal and reproductive disorders, including changes in the testicles. In addition to obesity, exposure to endocrine disruptors (ED) and other environmental and behavioral factors contribute to the development of these diseases (4, 5).

The ED are defined as a chemical compound, or a mixture of compounds, that interfere with any aspect of hormonal action (6), with a wide variety of substances that are considered DE, such as dichloro-diphenyl-trichloroethane (DDT), bisphenol A (BPA), phthalates, parabens and dioxin (7). Due to its structural similarity to BPA, studies currently point to BPS as an ED (8).

BPA is a high-volume chemical commonly used in food packaging materials, dental sealants, medical devices, and thermal prescriptions, with bisphenol exposure being ubiquitous through ingestion, inhalation, and dermal contact (9), so it is found in generally in the form of plastics that are widely used by consumers for food storage (10).

Despite the widespread use of bisphenol in everyday consumer products, comprehensive understanding of its direct impacts on the testicles remains limited. This gap in knowledge is particularly relevant considering the growing concern about human exposure to endocrine-disrupting environmental chemicals. Given the crucial importance of the testicles for reproduction and the endocrine system, it is imperative to fill this gap. This study attempts to address this shortage, contributing to a more complete understanding of the potential effects of bisphenol on male reproductive health and providing a solid basis for future interventions and regulations.

Our hypothesis is that simultaneous exposure to a high-fat diet and BPS will cause testicular morphological changes. The aim of the work was to evaluate the morphological and stereological parameters of the testicles in mice of the C57BL/6 lineage exposed to Bisphenol S on a control diet and with induced obesity.

## MATERIALS AND METHODS

This study followed the International Guiding Principles for Biomedical Research involving animals, published by the Council for International Organizations of Medical Sciences (CIOMS), as well as with the Brazilian law on the scientific use of animals and was approved by the ethics committee for animal experimentation of our institutional committee (IRB number: 2504060718).

Forty adult male C57BL/6 mice were fed a standard diet (standard show, SC) or high-fat diet (HF) for a total of 12 weeks. From the beginning of the experimental protocol, each group was randomly divided into 4 experimental groups with 10 mices as follows: a) SC - animals fed a standard diet; b) SC-B - animals fed a standard diet and administration of BPS (25 μg/kg of body mass/day) in drinking water; c) HF: animals fed a high-fat diet; d) HF-B - animals fed a high-fat diet and administration of BPS (25 μg/Kg of body mass/day) in drinking water. BPS administration lasted 12 weeks, following exposure to the SC and HF diets. The BPS dose is according to NOAEL concentrations (No Observed Adverse Effect Level) for systemic toxicity from studies using rodents as a model (https://echa.europa.eu/). BPS was diluted in absolute ethanol (0.1%) and added to drinking water at a concentration of 25 μg/kg body weight/day.

At the end of the 12th week, the animals were fasted for 6 hours, heparinized and deeply anesthetized with a combination of ketamine (40 mg/kg) and xylazine (8 mg/kg) intraperitoneally, with the testicles removed for processing.

**Figure 1:**
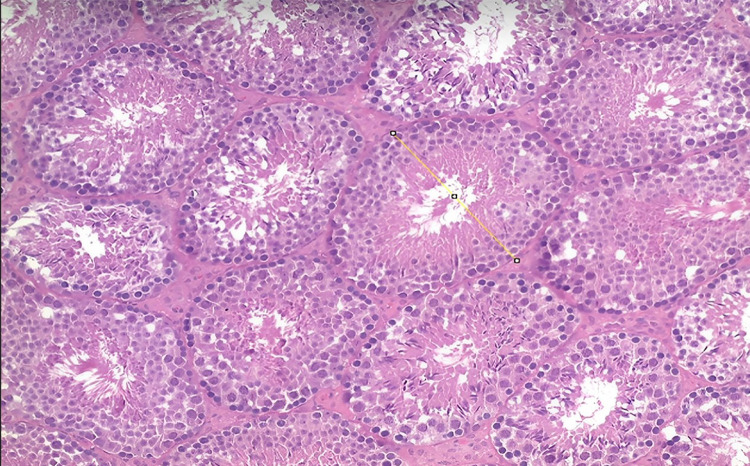
The figure shows the morphometric analysis of seminiferous tubules using the Image J software. The image was captured with an Olympus BX51 microscope and Olympus DP70 camera (Hematoxylin and Eosin (H&E) X100).

The testes were fixed in 10% buffered formalin, processed according to laboratory routine and subsequently embedded in paraffin. Sections with a thickness of 5 µm were then made and stained with hematoxylin and eosin (H&E). For stereological and morphometric analysis, 5 animals per group were used. The images were captured with an Olympus BX51 microscope and Olympus DP70 camera. The stereological analysis was done with the Image Pro and Image J programs (Figure-1).

Plasma was separated by centrifugation (120 g for 20 min) at room temperature and stored individually at -80ºC for further analysis. Plasma concentrations of total cholesterol and triglycerides were measured with commercially available colorimetric kits (Bioclin System II, MG, Brazil).

The mean diameter of the seminiferous tubules was measured in 5 animals per group, each animal owning 10 different fields, with 10 measurements performed, totaling 500 measurements per group. The measurements were performed regardless of the phase of the cycle of the seminiferous epithelium. Sections were chosen randomly by scanning horizontally and those that contained more circular possible outline were chosen. All the scanning was performed in an optical microscope with a 10X objective. Diameters were measured by image analysis using Image-Pro.

To measure the thickness of the tunica albuginea, 10 linear measurements were used, carried out in 5 different fields, and randomly distributed throughout the testicle in a final total of 250 measurements and expressed in micrometers (μ). The volume of the testis was measured in grams of the Scherle’s method (11). The density per area (QA) of the seminiferous tubules was calculated in consideration of the number of the seminiferous tubules in a frame of known area when they did not hit 2 consecutive lines of the system (forbidden lines). The system was produced with the STEPanizer web-based system (12). The density of length (Lvtubules, testicule) of the seminiferous tubules was measured as described previously, as the total length (L) of the tubules (13, 14).

### Statistical Analysis

The samples were tested for their distribution and normality by the Kolmogorov-Smirnov (K-S) test and the statistical significance was determined by the analysis of variance (ANOVA) followed by the Holm-Sidak post-test. The level of significance was determined as P ≤ .05 (GraphPad Prism version 6.01 for Windows).

## RESULTS

The body mass of the HF and HFB groups began to increase significantly from the first week after starting the diet and at the end of 12 weeks this increase was greater than 12% of the control groups. The SCB group showed a significant difference in body mass in relation to the SC, HF and HFB groups from the fourth week of the experiment onwards and this remained the same until the end of the experiment.

In relation to total cholesterol when compared to the SC group, the SCB group had an increase of 34% in relation (p = 0.0056), while the HF groups had an increase of 83% and HFB of 67% and both with p < 0.0001. The HF and HFB groups also showed a significant increase compared to the SCB group of 37% and 25% respectively with p = 0.0001. Data on the evolution of body mass cholesterol and triglycerides during the experimental period are shown in Table-1.

**Table 1:** The table show the general experiment data Data were expressed as mean ± standard deviation (n = 10/group). Significant differences between groups were identified when p value < 0.05, as determined by one-way ANOVA and Holm-Sidak post-test.

	SC	SC-B	HF	HF-B	P value
**Body Mass (g)**					
Initial	23.8 ± 1.0	24.6 ± 1.0	24.2 ± 1.2	24.6 ± 1.2	No differences
Final	28.4 ± 1.7	31.9 ± 2.8	43.0 ± 2.3	41.8 ± 2.7	SC vs SCB vs HF;HFB<0.001
**Total Cholesterol (mg/dL)**	97.7 ± 12.1	131.1 ± 27.6	179.5 ± 22.4	163.2 ± 26.8	SC vs. SCB 0.0056
					SC vs. HF<0.0001
					SC vs. HFB<0.0001
					SCB vs. HF 0.0001
					SCB vs. HFB 0.0056
**Thickness of the tunica albuginea (um)**	177.0 ± 71.0	214.0 ± 105.2	384.7 ± 117.4	231.9 ± 51.2	SC vs. HF <0.0001
					SC vs. HF-B 0.0274
					SC-B vs. HF <0.0001
					HF vs. HF-B <0.0001
**Diameter of the seminiferous tubule (um)**	331.5 ± 41.7	417.6 ± 110.1	402.9 ± 95.6	400.1 ± 67.2	SC vs. SC-B <0.0001
					SC vs. HF 0.0011
					SC vs. HF-B 0.0015
**Density of seminiferous tubules by area (mm^2^)**	12.6 ± 0.7	8.5 ± 0.9	7.5 ± 1.9	7.9 ± 0.3	SC vs. SC-B 0.0002
					SC vs. HF <0.0001
					SC vs. HF-B <0.0001
**Seminiferous tubule length density (mm/mm^3^)**	25.2 ± 1.4	17.1 ± 1.8	15.1 ± 3.8	15.9 ± 0.7	SC vs. SC-B 0.0002
					SC vs. HF <0.0001
					SC vs. HF-B <0.0001
**Total length of the seminiferous tubules (mm)**	2175 ± 396.4	1604 ± 191.8	1559 ± 440.9	1217 ± 226.3	SC vs. HF 0.0432
					SC vs. HF-B 0.0016

SC = standard diet; SCB = standard diet + BPS exposure; HF = high-fat diet; HFB = high-fat diet and BPS exposure.

**Figure 2:**
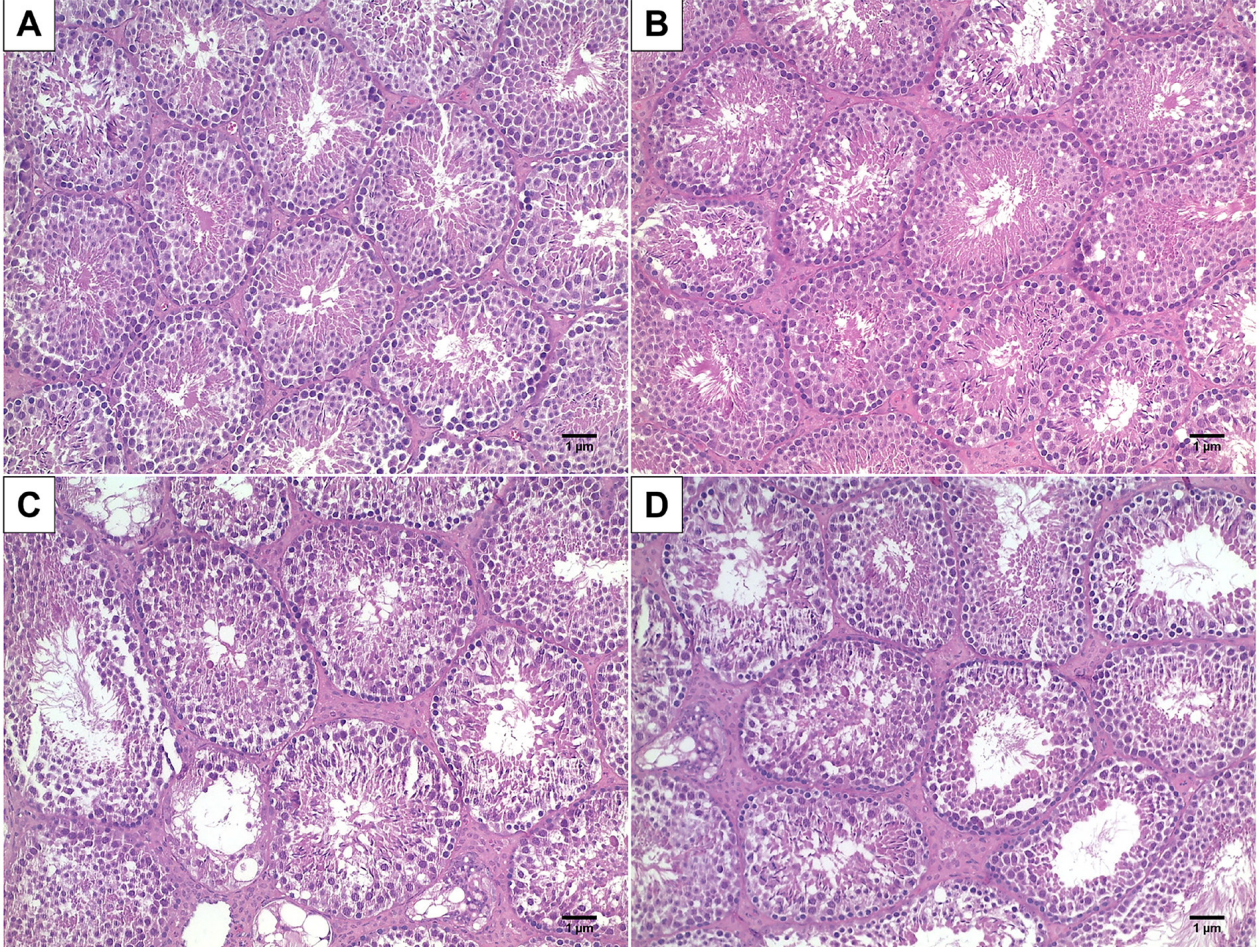
Seminiferous tubules Density.

**Figure 3:**
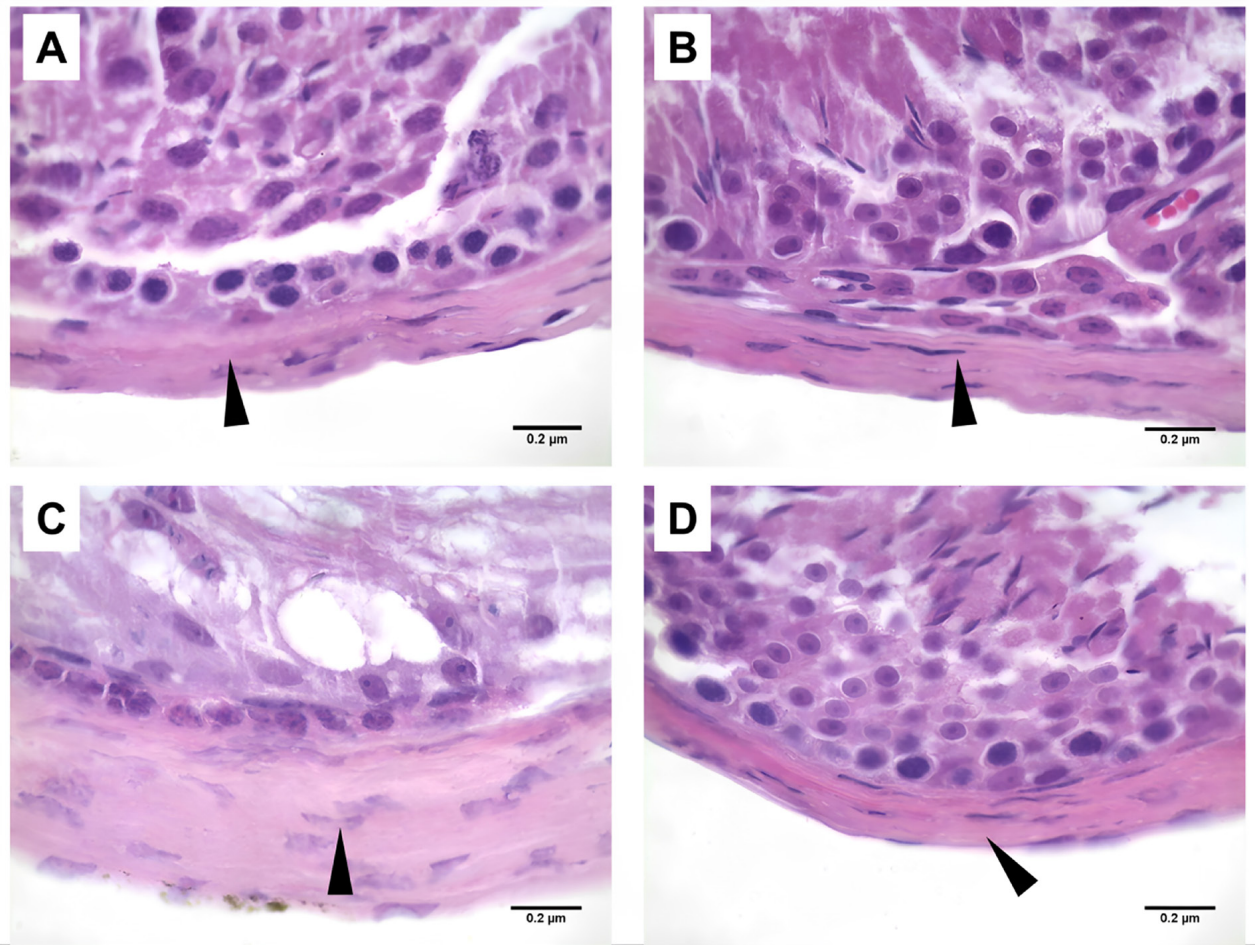
Tunica albuginea Thickness.

The seminiferous tubule density per area reduced in all groups when compared with SC samples (p<0.001): HF (40%), SC-B (32%), and HF-B (36%) (Figure-2 and Table-1). Length density was reduced significantly (p<0.001) in all groups when compared with SC group: HF (40%), SC-B (32%), and HF-B (36%). The seminiferous tubule total length was reduced (p<0.001) when compared to f HF (28%) and SC-B (26%) groups (Table-1). The tubule diameter increased significantly (p<0.001) only when we compared the SC group with SC (54%) and SC-B (25%) groups, and the tunica thickness increased significantly only in HF group (117%) when compared with SC-B (20%) and HF-B 31% (Figure-3 and Table-1).

A) Photomicrography showing the seminiferous tubules of SC group (animals fed a standard diet); B) Photomicrography showing the seminiferous tubules of SC-B group (animals that fed a standard diet and administration of BPS); C) Photomicrography showing the seminiferous tubules of HF group (animals that fed a high-fat diet) and D) Photomicrography showing the seminiferous tubules of HF-B group (animals that fed a high-fat diet and administration of BPS (H&E X100). The seminiferous tubule density per area reduced in all groups when compared with SC samples.

A) Photomicrography showing the tunica albuginea (arrowhead) of SC group (animals that fed a standard diet); B) Photomicrography showing the tunica albuginea (arrowhead) of SC-B group (animals that fed a standard diet and administration of BPS); C) Photomicrography showing the tunica albuginea (arrowhead) of HF group (animals that fed a high-fat diet) and D) Photomicrography showing the tunica albuginea (arrowhead) of HF-B group (animals that fed a high-fat diet and administration of BPS (H&E X100). The tunica thickness increased significantly only in HF group when compared with SC-B and HF-B.

## DISCUSSION

Obesity is one of the main risk factors for numerous non-communicable diseases, such as coronary heart disease, high blood pressure, stroke, certain types of cancer, type 2 diabetes, in addition to representing one of the main causes of secondary hypogonadism in men. This is characterized by impairment of the hypothalamic-pituitary-testicles (HPT) axis, in which the reduction in testosterone levels is also accompanied by signs and symptoms of hypogonadism, such as decreased libido, erectile dysfunction, semen quality, strength and mood (1, 15).

Bisphenols are a group of chemical compounds that contain two phenyl rings connected together by a small linking group. Bisphenol A (BPA) was discovered in 1891, described as belonging to the class of endocrine disrupting compounds (EDCs), substances that can effectively alter the endocrine system. Structurally, BPA is similar to estradiol and, consequently, ends up interfering with steroid signaling with different results on reproductive health, depending on doses, stage of life, mode and moment of exposure. And for a long time it was the most used in the industry and it is estimated that in 2015 the production of BPA was approximately 4.85 million tons, where 90% of this production was used in the manufacture of polycarbonates and epoxy-phenolic resins (9, 16, 17).

Faced with numerous limitations on BPA from regulatory agencies, different alternative plasticizers to BPA have been developed, such as BPS, bisphenol B, bisphenol F and bisphenol AF, with BPS being the main substitute for BPA (18, 19). It has recently been described that exposure to BPA causes numerous health impacts. The risk posed by exposure to this compound is still poorly assessed, especially in small or low doses. A few available data associate BPS with several conditions, suggesting that it is equally or even more toxic than BPA. Bisphenol S (BPS), which is widely used in the production of plastics and can be found in a variety of everyday products is associated with testis toxicity (20-23). In the present paper we studied the influence of the high fat diet and the exposure to BPS in the same experiment.

It has recently been described that exposure to BPA causes numerous health impacts. In the reproductive system, this ED is associated with reduced libido, changes in sperm count and quality, polycystic ovary syndrome, precocious puberty, among several other effects (20). In the reproductive system, effects such as decreased fertility and the rate of embryonic implantation have already been described, in addition to toxicity for the nervous and immune systems and association with the development of cancer and type 2 diabetes mellitus (8, 21-23).

Exposure to BPA was associated with decreased sperm concentrations and impaired sperm parameters, as well as a raised percentage of immature sperm and reduced testosterone levels (24). In a nice previous study the authors shows that exposure of male rats to BPA and is analogs BPB, BPF, and BPS resulted in decreased sperm production, testosterone secretion, and histological changes in the reproductive tissues (25). Histological parameters of both testis and epididymis revealed prominent changes in the reproductive tissues in cases of BPS exposition (26). Our results are similar and suggest that BPS exposition led to marked alterations in the development of the male reproductive system. We observed that the seminiferous tubule morphological parameters were significantly reduced in rats exposed to BPS.

Reproductive dysfunction is a common consequence of obesity (27). The high fat diet had a negative impact in male rat fertility (28). Some studies show that the high fat diet correlates with irreversible changes in testis metabolism, steroidogenesis, germ cell proliferation, testis histology and apoptosis (27, 29). The high fat diet induces modifications in the testes structure and sperm parameters in rats (30). In our sample we observed that the mice submitted to high fat diet had important alterations in testis. The high fat diet leads to seminiferous tubule diameter, length and density significant reduction when comparing with the control group which confirms previous studies about this topic (30).

This work has some limitations that should be highlighted: (a) the sample size was small; (b) In our study, we used an animal model within controlled experimental conditions; therefore, the results of this study may not represent what occurs in the scenario of exposure to BPA and (c) dietary and environmental components can interact with Bisphenol S.

## CONCLUSIONS

Animals exposed to bisphenol S and/or high-fat diet-induced obesity presented important structural alterations in testicular morphology which suggest that this condition causes a deleterious effect in male genital system.

**Capsule:** Animals exposed to bisphenol S and/or high-fat diet-induced obesity presented important structural alterations in testicular morphology which suggest that this condition causes a deleterious effect in male genital system.
